# [1-^11^C]-Butanol Positron Emission Tomography reveals an impaired brain to nasal turbinates pathway in aging amyloid positive subjects

**DOI:** 10.1186/s12987-024-00530-y

**Published:** 2024-04-02

**Authors:** Neel H. Mehta, Xiuyuan Wang, Samantha A. Keil, Ke Xi, Liangdong Zhou, Kevin Lee, Wanbin Tan, Edward Spector, Amirhossein Goldan, James Kelly, Nicolas A. Karakatsanis, P. David Mozley, Sadek Nehmeh, J. Levi Chazen, Simon Morin, John Babich, Jana Ivanidze, Silky Pahlajani, Emily B. Tanzi, Leslie Saint-Louis, Tracy Butler, Kewei Chen, Henry Rusinek, Roxana O. Carare, Yi Li, Gloria C. Chiang, Mony J. de Leon

**Affiliations:** 1 Department of Radiology, Brain Health Imaging Institute, Weill Cornell Medicine, 407 East 61 Street, 10065 New York, NY USA; 2 Harvard Medical School, Boston, MA USA; 3 Department of Radiology, Molecule Imaging Innovations Institute, Weill Cornell Medicine, New York, NY USA; 4 Radiopharm Theranostics, New York, NY USA; 5 Department of Radiology, Hospital for Special Surgery, New York, NY USA; 6 Department of Radiology, NYU Langone Health, New York, NY USA; 7 Lenox Hill Radiology, New York, NY USA; 8 Faculty of Medicine, University of Southampton, Southampton, UK; 9 Department of Radiology, Weill Cornell Medicine, New York, NY USA; 10 Weill Cornell Medicine, School of Medicine New York, New York, NY USA; 11 Ratio Therapeutics, Boston Mass, USA; 12 University of Michigan, Ann Arbor, MI USA; 13 College of Health Solutions, Arizona State University, Downtown Phoenix Campus, Arizona, USA

**Keywords:** Dynamic PET, [1-11 C]-Butanol, CSF clearance, Glymphatic, Amyloid PET, Nasal turbinates, Cribriform plate, Alzheimer Disease, Aging

## Abstract

**Background:**

Reduced clearance of cerebrospinal fluid (CSF) has been suggested as a pathological feature of Alzheimer’s disease (AD). With extensive documentation in non-human mammals and contradictory human neuroimaging data it remains unknown whether the nasal mucosa is a CSF drainage site in humans. Here, we used dynamic PET with [1-^11^C]-Butanol, a highly permeable radiotracer with no appreciable brain binding, to test the hypothesis that tracer drainage from the nasal pathway reflects CSF drainage from brain. As a test of the hypothesis, we examined whether brain and nasal fluid drainage times were correlated and affected by brain amyloid.

**Methods:**

24 cognitively normal subjects (≥ 65 years) were dynamically PET imaged for 60 min. using [1-^11^C]-Butanol. Imaging with either [^11^C]-PiB or [^18^F]-FBB identified 8 amyloid PET positive (Aβ+) and 16 Aβ- subjects. MRI-determined regions of interest (ROI) included: the carotid artery, the lateral orbitofrontal (LOF) brain, the cribriform plate, and an All-turbinate region comprised of the superior, middle, and inferior turbinates. The bilateral temporalis muscle and jugular veins served as control regions. Regional time-activity were used to model tracer influx, egress, and AUC.

**Results:**

LOF and All-turbinate 60 min AUC were positively associated, thus suggesting a connection between the brain and the nose. Further, the Aβ+ subgroup demonstrated impaired tracer kinetics, marked by reduced tracer influx and slower egress.

**Conclusion:**

The data show that tracer kinetics for brain and nasal turbinates are related to each other and both reflect the amyloid status of the brain. As such, these data add to evidence that the nasal pathway is a potential CSF drainage site in humans. These data warrant further investigation of brain and nasal contributions to protein clearance in neurodegenerative disease.

**Supplementary Information:**

The online version contains supplementary material available at 10.1186/s12987-024-00530-y.

## Background

Recent observations from human neuroimaging studies and animal models have identified impaired cerebrospinal fluid (CSF) clearance in the progressive development of Alzheimer’s Disease (AD) [1–8]. AD is characterized by the accumulation of beta-amyloid (Aβ) [9] and tau proteins [10, 11], which are partially attributed to a failure of brain waste clearance mechanisms [5, 12]. Predominantly derived from the choroid plexus of the brain ventricular system, CSF is produced via the passive and active filtration of blood [13]. In addition to providing hydrostatic protection and nutrients to the brain and spinal cord, when mixed with interstitial solutes and metabolites, CSF provides the vehicle for clearing toxic proteins and metabolic byproducts, a necessary function regulating nervous system health [12, 14].

While a large body of literature has reported the existence of CSF drainage through the cribriform plate and nasal turbinates in animal models [15–18], the existence of a nasal CSF clearance pathway in humans remains controversial [19]. As we recently reviewed [18], the brain-nose junction has the potential for both non-invasive diagnosis and therapeutic interventions in neurodegenerative illnesses. However, with only limited post-mortem [20] and in-vivo tissue biopsy studies [1, 21] there remains uncertainty whether the nasal region is involved in human CSF clearance. Here, we use the highly permeable and non-binding [1-^11^C]-Butanol PET tracer, which enters and clears the blood and brain to examine carotid artery, brain, and nasal pathway tracer influx and drainage. We tested two hypotheses: that brain and nose tracer kinetics are associated, and that the presence of cerebral amyloid impairs tracer kinetics in both brain and nose.

## Methods

### Study participants

24 elderly independent community residing volunteers (mean age: 75.1 ± 6.4 years, 10 male and 14 female) were recruited into this IRB approved study at Brain Health Imaging Institute, Weill Cornell’s Department of Radiology. All subjects underwent standardized clinical assessment, MRI, ApoE genotyping for (Ɛ4 + or Ɛ4-) carrier status, [1-^11^C]-Butanol for quantitative clearance estimates, and either [^11^C]-PiB (*n* = 21) or [^18^F]-FBB (*n* = 3) PET for amyloid (Aβ+ or Aβ-) status. Executive and memory functions were assessed using the Clinical Dementia Rating (CDR) [22], the Global Deterioration Score (GDS) [23], the Rey Auditory Verbal Learning Total Recall-Delayed (RAVLT) [24], and the Craft Story 21 Recall-Delayed [25]. Subject diagnoses were made in conjunction with National Institute of Neurological and Communicative Disorders and Stroke/AD and Related Disorders Association criteria through consensus conferences involving neurologists, neuroradiologists, and neuropsychologists [26]. Subjects with nasal pathway disease or surgery (imaging and self-report) or expressing cognitive impairment as determined by a CDR > 0 or GDS > 2 were excluded. For hypothesis testing, 8 subjects were classified as Aβ+ and 16 as Aβ-.

### Image acquisitions

#### MRI

Subjects underwent a 3T SIEMENS MAGNETOM Prisma MRI scan with a 64-channel head/neck coil. The MRI protocol included a T1-weighted (T1W) MPRAGE sequence (TI = 900ms, TR/TE = 2400/2.96ms, flip angle = 9, voxel size = 0.5 × 0.5 × 0.5mm^3^, voxel dimension = 512 × 512 × 416) and a T2-weighted (T2W) SPACE sequence (Echo train duration = 896ms, TR/TE = 3200/408ms, flip angle = 120, voxel size = 0.5 × 0.5 × 0.5mm^3^, matrix = 512 × 512 × 320). The T2W-SPACE sequence was used for improved definition of the pial surface and for anatomical sampling of the turbinates and cribriform plate regions of interest (ROI).

#### PET

Each subject received Butanol and amyloid PET scans run on 2 separate days with a mean interval of 3 months. PET tracers were intravenously administered with the average doses for [1-^11^C]-Butanol (480MBq), [^11^C]-PiB (464MBq) or [^18^F]-FBB (301MBq). Subjects were scanned using the same Biograph64_mCT scanner for PET images, which were acquired in list mode and reconstructed with attenuation and decay corrections. [1-^11^C]-Butanol was delivered intravenously as a bolus over approximately 10s (average injection volume: 5.43±3.03 ml). Dynamic acquisitions began with the injection and continued for 60 min. The data were reconstructed into 45 frames (18 × 10s, 4 × 30s, 15 × 60s, 8 × 300s) using a 512 × 512 × 111 matrix, resulting in a voxel size 0.8 × 0.8 × 2mm^3^. [^11^C]-PiB PET data were acquired between 40 and 90 min after injection and reconstructed into five 10 min frames, with a 512 × 512 × 74 image matrix and a voxel size 0.8 × 0.8 × 3mm^3^. Three subjects received [^18^F]-FBB instead of [^11^C]-PiB. The [^18^F]-FBB PET data were acquired from 90 to 110 min after injection and reconstructed into four 5 min frames. The image size was 400 × 400 × 74 and the voxel size was 1 × 1 × 3mm^3^. All images were acquired in the supine position.

### Image processing

#### MRI

The T1W-MPRAGE scan was processed for anatomical segmentation and surface reconstruction using Freesurfer v7.1 [27]. The T2W-SPACE scan was used to optimize identification of the pial surface by using T2W image as the secondary input. Both T1W and T2W images were formatted into 1 mm isotropic 256 × 256 × 256 voxel space after Freesurfer processing.

#### PET

For the [1^-11^C]-Butanol PET scan, the time frames were first realigned to the average image derived from the 30 s to 5 min time frames using FSL MCFLIRT (Motion Correction FLIRT) [28]. The realigned frames were then co-registered to the Freesurfer processed T1W scans with rigid transformation using FSL FLIRT [29]. Linear interpolation was used to resample the regional 0 to 60 min time activity curves (TACs) at 10 s intervals. All calculations using [1-^11^C]-Butanol tracer signal were body weight and dose adjusted standardized uptake values (SUV).

The [^11^C]-PiB and [^18^F]-FBB PET scans went through a similar realignment and co-registration process using the mean image from 40 to 90 min for [^11^C]-PiB as the realignment template and 90 to 110 min for [^18^F]-FBB. To assist the clinical amyloid readings, voxel wise standardized uptake value ratios (SUVR) were calculated with reference to the cerebellar cortex gray matter using the 60 to 90 min [^11^C]-PiB data [30], and 90 to 110 min for the [^18^F]-FBB data. All amyloid PET images were read by a highly experienced board-certified neuroradiologist. The Aβ diagnosis was given if there was evidence of binding in the neocortex, posterior cingulate, or precuneus. The amyloid diagnosis was conducted blind to the study results.

### Region of interest segmentation

Carotid artery, brain, nasal compartment, and control regions were selected to characterize tracer influx and egress.

#### The nasal compartment

**Fig. 1:**
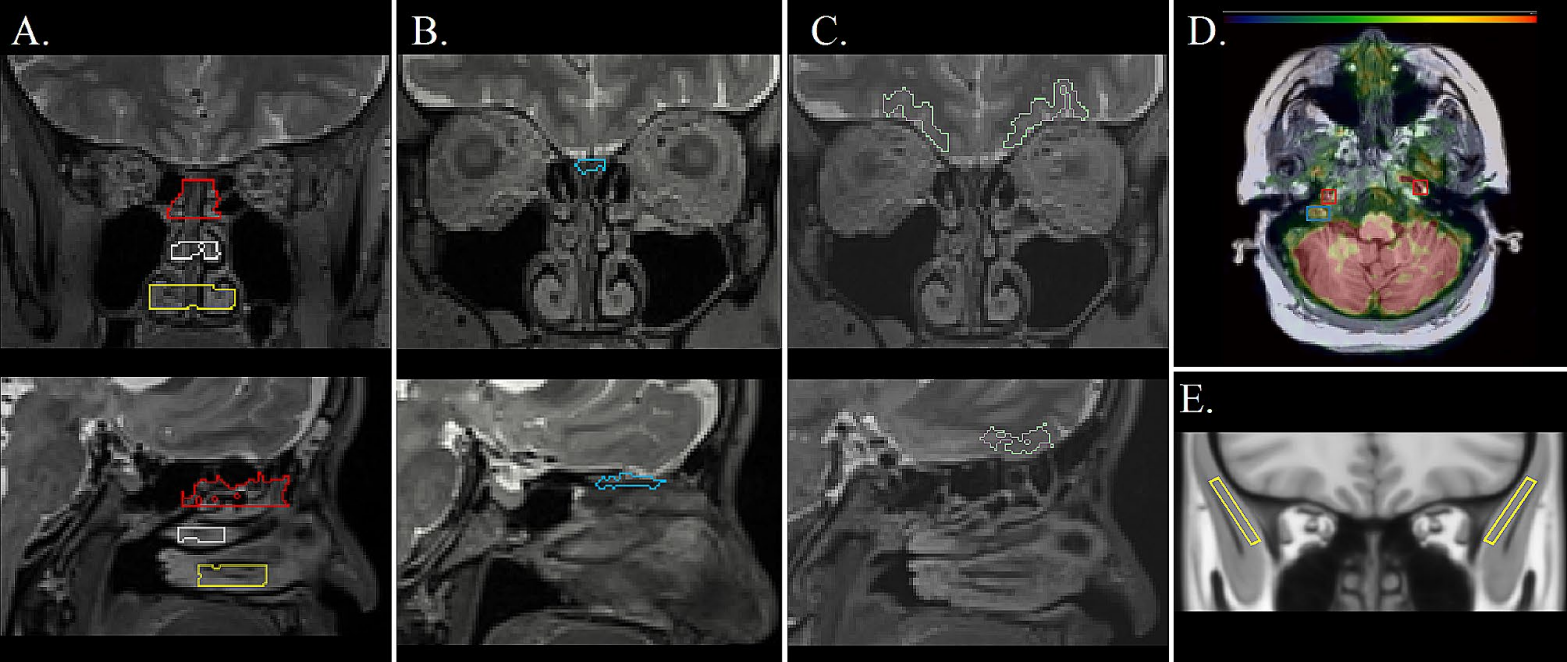
Defining Regions of Interest. Coronal (top row) and sagittal (bottom row) view of ROIs in **(A)** superior (red), middle (white), inferior (yellow) turbinates displayed on T2W-MRI; **(B)** the cribriform plate outline (blue) on T2W-MRI; **(C)** the Freesurfer lateral orbitofrontal (LOF) cortex (green) on T2W-MRI; **(D)** [1-^11^C]-Butanol PET at 40s post injection displaying on T1W-MRI the location of the seeds for left and right internal carotid artery (red boxes) and right jugular vein (blue box) that were input into vessel-tracking algorithms to generate three-dimensional ROIs; and **(E)** the temporalis muscle ROI (yellow) was sampled on the MNI152_T1 template

Four nasal pathway ROIs were evaluated in each participant including the cribriform plate, and the superior, middle, and inferior turbinates (Fig. 1A-B). For accurate anatomical identification of the three turbinates, each subject’s Freesurfer processed T2W image, followed an adapted anterior and posterior commissure (AC-PC) alignment method from the Human Connectome Project preprocessing pipeline [31]. Segmentation of each turbinate was performed in a semi-automatic way using ITK-SNAP [32]. A bounding box was manually placed for the individual turbinates in the AC-PC aligned T2W image. A high-pass filter thresholded out the lowest quartile of the voxel intensities removing the air-like voxels from the turbinate tissues. Circular seeds with 1.5-3 mm diameter were placed in various regions of the turbinate tissues, growing over nearby tissues across 10,000 cycles. This minimized air-filled spaces for each turbinate ROI (Fig. 1A). The final ROIs were linearly transformed to Freesurfer T1W space by inverse AC-PC alignment to match the co-registered PET images. A combined nasal turbinate ROI (All-turbinates) which consisted of the three turbinate ROIs described above was also evaluated.

A cribriform plate ROI was included. Due to its small size, orientation, and between subject variability [33, 34], each subject’s cribriform plate was manually drawn as an ROI on sagittal T1W and T2W slices with precise adjustments made using axial and coronal views. Using the olfactory sulci and the olfactory bulbs as landmarks, a maximum 20 mm long (anterior-posterior), 10 mm wide (left-right) and 5 mm high (superior-inferior) ROI was placed on the cribriform plate (Fig. 1B). All regions of interest were examined and agreed to by two expert anatomists.

Bilateral ROIs of the temporalis muscle were selected as an extracranial control region due to its lack of involvement in lymphatic drainage from the brain. The ROIs were manually drawn based on the Montreal Neurological Institute (MNI) standard space [35] (Fig. 1E). The non-linear warping field from the individual T1W-MPRAGE to MNI152 space was done using Advanced Neuroimaging Tools [36] and the inverse field was applied to the ROIs to match the subject space.

#### Vascular anatomy

To evaluate and control for blood borne tracer contributions to tracer egress effects, ROIs were defined for the carotid artery and jugular vein as implemented in FireVoxel software, build 431 A (https://firevoxel.org/) following the procedures described in Mikheev et al. [37]. This semi-automated technique included a two-stage approach of manual placement of a seed (Fig. 1D), and subsequent automated vessel tracking [37]. The internal carotid seed was placed on axial PET slices after locating on MRI the petrous segment (C2) of the artery. High intensity PET signal reflecting venous tracer drainage was used to place the jugular seed between the internal jugular vein and sigmoid sinus. Vessel tracking parameters were set to a maximum diameter of 3 mm with a maximum length of 10 mm.

#### Brain anatomy

We sampled bilaterally using Freesurfer the auto-segmented LOF cortex (see Fig. 1C). The LOF is a cerebral gray matter region close to the cribriform plate. It was chosen to investigate the hypothesized fluid connection between brain and nasal compartments.

#### Assessment of tracer influx and egress

**Fig. 2:**
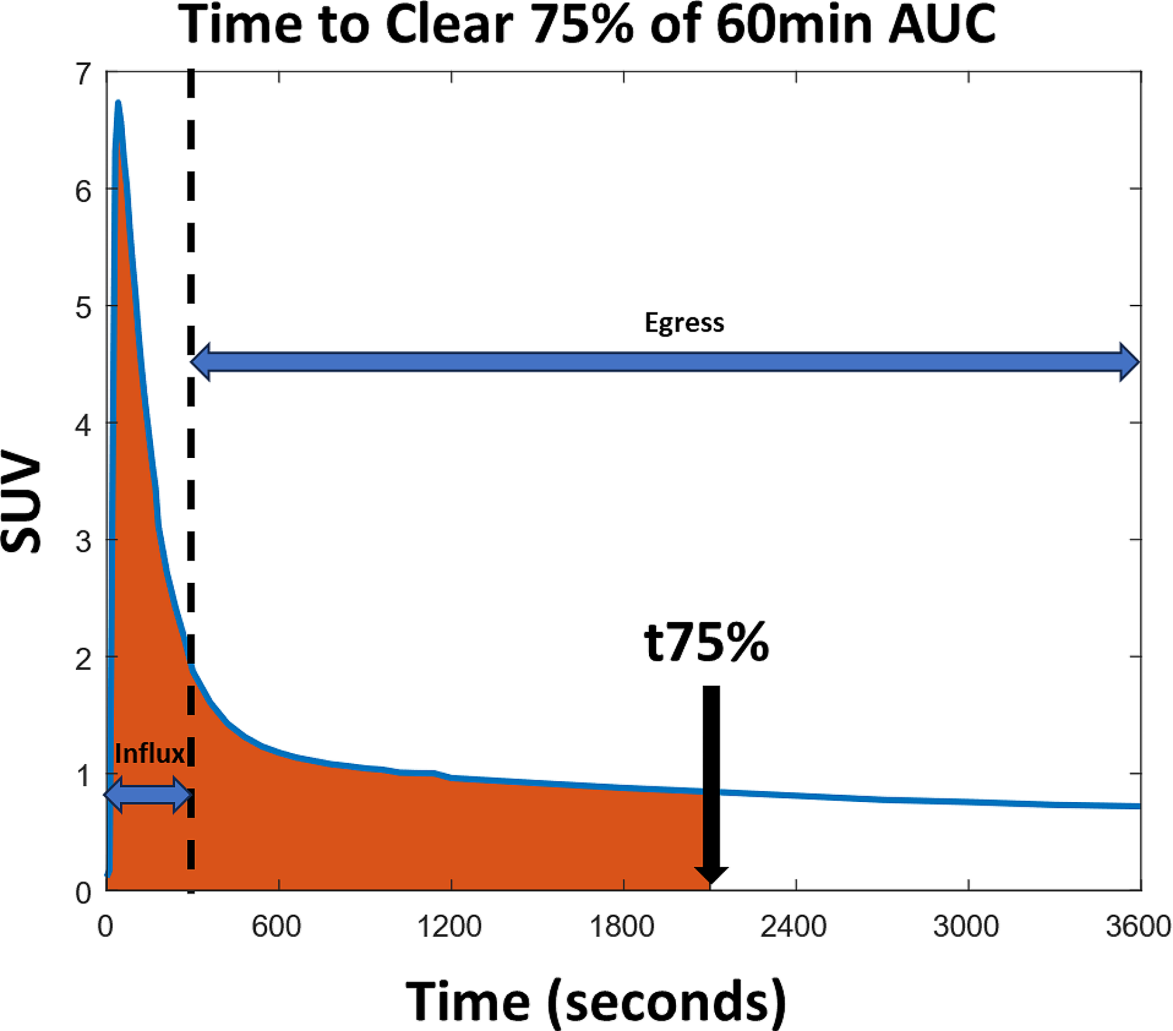
Schematic definition of t75%. A theoretical regional time-activity curve showing a time in seconds at which 75% of the tracer exposure (orange) is cleared (t75%). The black dashed line divides the TAC into 0-5 min influx and 5-60 min egress segments. The tracer concentration is expressed in SUV

Areas Under the Curve (AUC) using the trapezoid method [38] were calculated from the Butanol PET using the 0–60 min SUV-TAC. Brain and nasal tissue TACs were divided into 0 to 5 min influx segments where the blood is the major source of the signal and into a 5 to 60 min egress segment to maximize tracer egress. For the carotid artery, the TAC was segmented at 30 s as all subjects showed peak times under 1 min. Additionally, for brain and nasal tissues, we averaged the time in seconds for the tracer to fill and clear 75% of the 60 min AUC (t75%, see Fig. 2). The t75% was defined as: ${\int }_{0}^{t75\%}C\left(t\right)dt=0.75{\int }_{0}^{60min}C\left(t\right)dt$

The right side upper integral limit is the 60 min. acquisition time.

### Statistical analysis

Statistical analyses were performed using R studio (Version 2023.03.0 + 386.) and Prism (version 9.5). Continuous measures like age and cognitive variables (CDR, GDS, CRAFT, RAVLT) were assessed across dichotomized Aβ and Ɛ4 subgroups using Mann-Whitney test. Dichotomous frequency differences (Aβ subgroups, sex and ApoE Ɛ4 carrier status) were evaluated with chi square (χ2) analyses.

For measures that were not normally distributed (Shapiro-Wilk test), non-parametric Wilcoxon rank sum tests were used to examine Aβ subgroup differences. Multiple regression models were used to predict amyloid subgroup after controlling for confounds (carotid artery and jugular vein t75%). The false discovery rate (FDR) was used to correct for multiple comparisons [39, 40]. Two-Way Repeated Measure ANOVAs with the Geisser-Greenhouse correction for the violation of sphericity were run for the LOF and the All-turbinates to examine the main effects of Aβ subgroup and time, and their interaction on the [1-^11^C]-Butanol TAC. Follow-up Two-Way ANOVAs were run to examine the interactions for tracer influx and egress segments. Statistical analyses were performed on TACs interpolated to a 10 s interval across 60 min to avoid biasing results from oversampling the more frequent early intervals. For all results, statistical significance was defined as a two-sided *p*-value ≤ 0.05.

## Results

### Clinical and demographic characteristics of the study participants

A total of 24 subjects were studied (mean age 75.1 +/- 6.4 years; see demographic data in Table 1**)**. The sample included 14 women [58.3%]; 8 Aβ+ [33%]; and 9 ApoE Ɛ4 carrier [37.5%]. Clinical readings of the [^11^C]-PiB PET and [^18^F]-FBB PET identified 16 subjects as Aβ- including two scanned with [^18^F]-FBB) and 8 Aβ+ subjects including one scanned with [^18^F]-FBB.

There were no significant differences in age, gender, or cognitive performance between Aβ subgroups. A significantly increased weight was observed in the Aβ- subgroup. As expected, the ApoE Ɛ4 carriers showed a greater frequency of Aβ+ subjects (see Supplementary Table 1).

**Table 1:** Demographics stratified by Aβ+/- status *Indicates significant difference between groups at *p* ≤ 0.05. Abbreviations: Interquartile range (IQR), median (md), Clinical Dementia Rating (CDR) [22], Global Deterioration Scale (GDS) [23] Rey Auditory Verbal Learning Total Recall-Delayed (RAVLT) [24], Craft Story 21 Recall-Delayed (CRAFT) [25]. No age, gender or cognitive differences were observed between the Aβ subgroups. ApoE Ɛ4 carriers were overrepresented in the Aβ + subgroup (*p* < 0.001)

Demographic Characteristics				
	Aβ - (*n* = 16)	Aβ + (*n* = 8)	Statistical Test	*p*
Age– Mean (SD) [Range]	76.1 (7.2) [65–86]	73.3 (4.3) [66–78]	Mann-Whitney	0.35
Gender– Female/Male	9 /7	5/3	chi square	0.78
Weight (kg)–Mean (SD) [Range]	76.7 (15.8) [54–121]	59.8 (8.52) [48–72]	Mann-Whitney	0.00451*
ApoE Ɛ4 (- /+)	14/2	1/7	chi square	< 0.001*
**Cognitive Measures**				
CDR md	0	0	Mann-Whitney	0.99
GDS md	2	2	Mann-Whitney	0.98
CRAFT md (IQR)	14.5 (5.5)	16 (5.0)	Mann-Whitney	0.21
RAVLT md (IQR)	8 (6.5)	11 (6.5)	Mann-Whitney	0.66

### Tracer kinetic relationships between brain and nasal turbinates

**Fig. 3:**
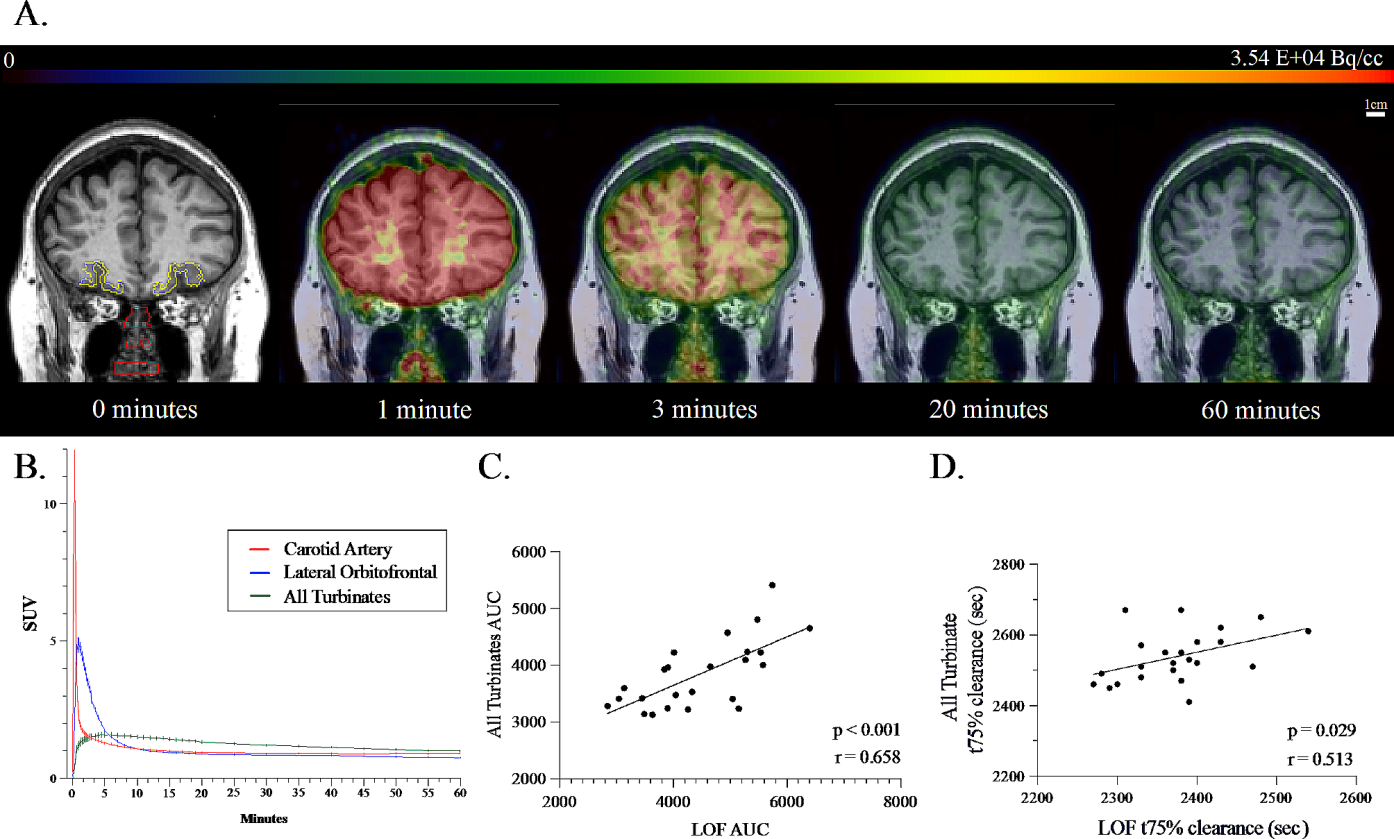
[1-^11^C]-Butanol Clearance **(A)** [1-^11^C]-Butanol PET images overlayed on coronal T1W-MRI showing for a representative subject the brain and nasal tracer concentrations at 0, 1 min, 3 min, 20 and 60 min after tracer administration. The nasal turbinate ROI (red) and LOF cortex (yellow) are displayed at 0 min. Scale bar (1 cm) is displayed for image size, and color bar is presented in radiotracer concentration (Bq/cc). **(B)** For the entire sample (*n* = 24), the mean and standard error of the Butanol time activity curves for the Carotid Artery (red), LOF (blue) and the All-turbinates (green). **(C)** The significant positive correlation between the 0-60 min AUC for LOF and All Turbinate tracer concentration (Spearman correlation *r* = 0.658, *p* < 0.001). **(D)** The significant positive correlation between the LOF t75% clearance time and the All turbinate t75% clearance time (Spearman correlation *r* = 0.513, *p* = 0.029)

Given the previous animal and limited human research supporting the existence of CSF egress from the brain through the nasal turbinates [1, 15, 18, 20, 21], we examined the average brain and nasal influx and clearance of [1-^11^C]-Butanol over 60 min (Fig. 3A). The TACs for the LOF and All-turbinates are found in Fig. 3B. A significant relationship between the LOF and the All-turbinates was observed for the 0-60 min AUC (Fig. 3C). Similarly, there was a significant relationship between the LOF and All Turbinates for the t75% (Fig. 3D). This brain to periphery relationship was not found between the LOF and either the temporalis muscle or the jugular vein.

### The effect of amyloid on brain and nasal turbinate tracer influx and egress

**Fig. 4:**
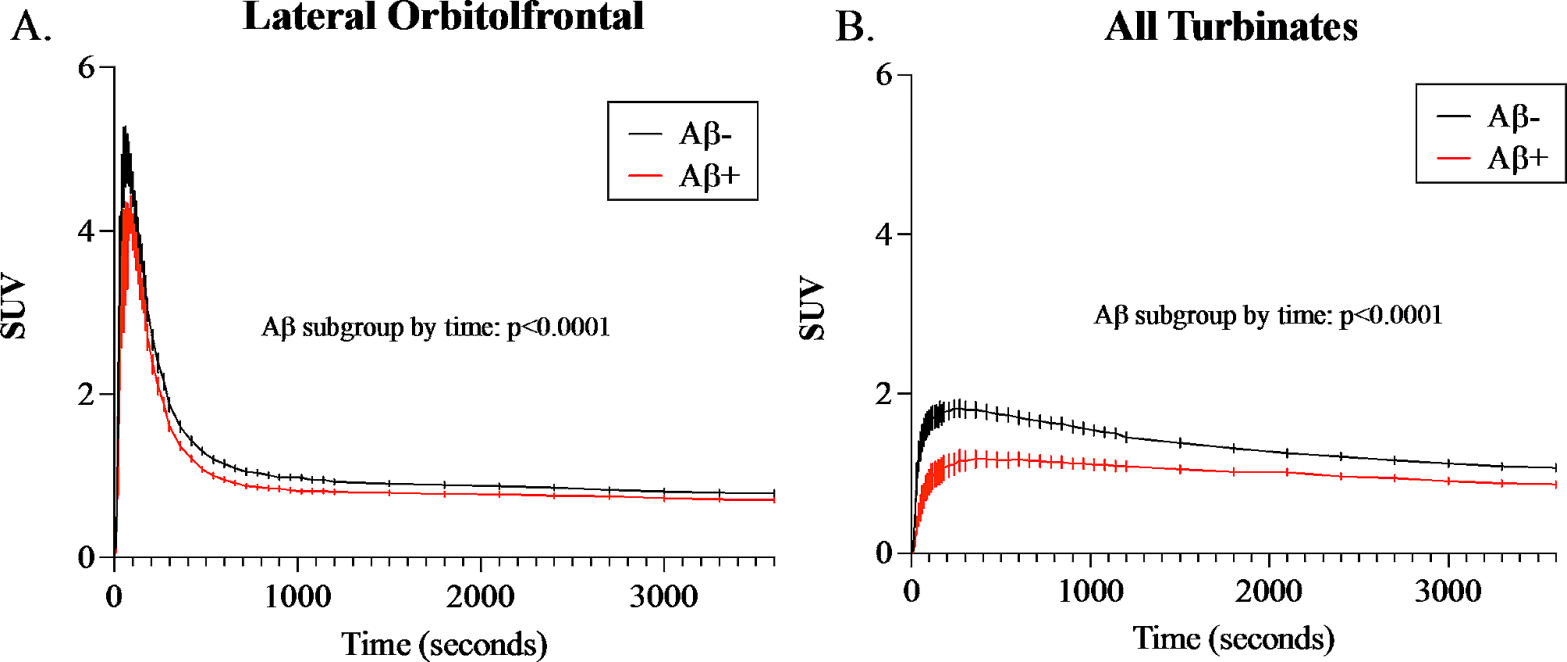
Reduced turbinate tracer input and egress in amyloid positive subjects. TACs from 0 to 60 min (3,600 s) following [1-^11^C]-Butanol administration. The Y-axis SUV is the radiotracer concentration normalized by dose and weight for: **(A)** LOF cortex and **(B)** All-turbinates. The LOF Repeated Measures Two-Way ANOVA showed a main effect of Aβ subgroup (F(1,22) = 4.587, *p* = 0.0435) and Aβ subgroup by time interaction (F(359,7898) = 1.453, *p* < 0.0001). Similarly, the All-turbinates showed a significant main effect for Aβ subgroup (F(1,22) = 11.29, *p* = 0.0023) and the Aβ subgroup by time interaction (F(359, 7898) = 6.684, *p* < 0.001)

To evaluate the effects of cerebral amyloid deposits on LOF and All-turbinate kinetics, we evaluated the [1-^11^C]-Butanol SUV-TAC curves across Aβ subgroups (Fig. 4). Repeated Measures Two-Way ANOVA of the 0-60 min SUV-TAC showed for the LOF a significant main effect for Aβ subgroup and a significant Aβ subgroup by time interaction. This demonstrated reduced brain tracer influx and slowed tracer egress in the Aβ+ subgroup (Fig. 4A). Similarly, the All-turbinates (Fig. 4B) showed a significant main effect for Aβ subgroup and Aβ subgroup by time interaction. Together, these data support the view that brain amyloid status is associated with reduced overall tracer kinetics in brain and nasal turbinates.

**Fig. 5:**
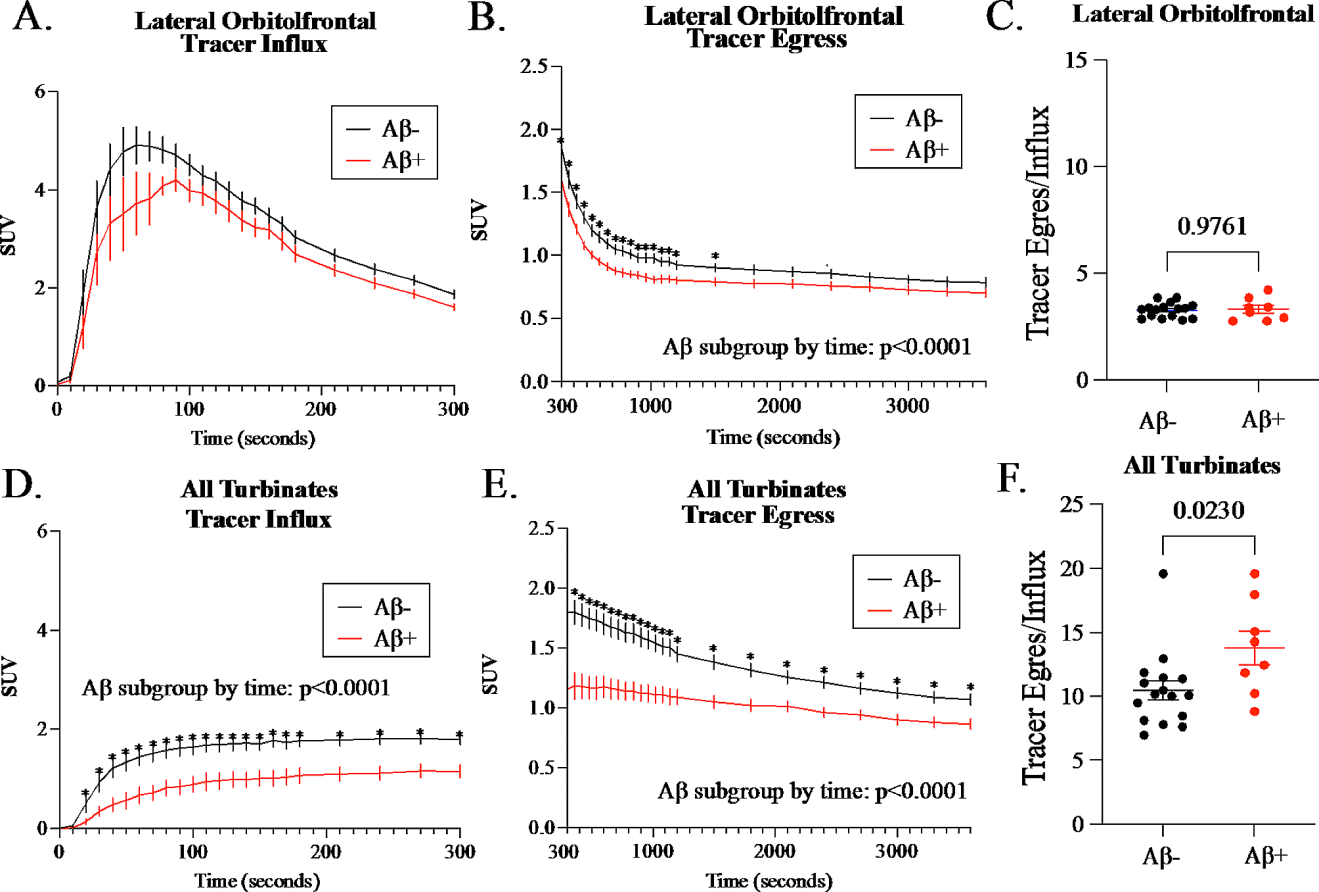
PET Butanol Influx and Egress in Lateral Orbitofrontal Cortex and All-turbinates. The effect of brain amyloid positivity on PET Butanol (SUV) influx and egress for both the LOF **(A-C)** and All-turbinates **(D-F).** The regional influx TAC from 0-5 min is seen in **(A, D)** and for the egress the TAC 5-60 min **(B, E). (A-B)** Using a Repeated Measures Two-Way ANOVA, the LOF influx showed a Aβ subgroup trend (F(1,22) = 3.16, *p* = 0.0892), and LOF egress showed a main effect of Aβ subgroup (F(1,22) = 4.641, *p* = 0.0424) and Aβ subgroup by time interaction (F(329, 7238) = 4.964, *p* < 0.0001). (D-E). For the influx to the All-turbinates, there was a main effect of Aβ subgroup and Aβ subgroup by time interaction: (F(1,22) = 10.24, *p* = 0.0041 and F(30,660) = 2.975, *p* < 0.0001, respectively) and for the egress from the All-turbinates: (F(1,22) = 11.36, *p* = 0.0028 and F(329, 7238) = 11.67, *p* < 0.0001, respectively). To assess the relative contributions of influx on egress within LOF and All-turbinates, the egress AUC was normalized by influx AUC (C, F). Mann-Whitney assessment of the normalized egress showed for the All-turbinates a significantly higher tracer ratio in Aβ + subjects. This supported the interpretation that for the Aβ + subgroup, impaired tracer egress from brain contributes in part to reduced turbinate tracer influx. Aβ- individuals are displayed in black, and Aβ + in red. Error bars represent the standard error of the mean on each time frame. FDR corrected significant differences at specific timepoints are denoted by *

To assess whether the Aβ associated differences in [1-^11^C]-Butanol kinetics were due to tracer influx or egress, we segmented the LOF and All-turbinate TACs into a tracer influx segment (0–5 min) and a tracer egress segment (5 to 60 min, see Fig. 5**)**. Interestingly, for the LOF, only the egress but not the influx, showed a significant Aβ subgroup main effect and the Aβ subgroup by time interaction (Fig. 5B). By comparison, the All-turbinates showed Aβ+ subgroup reductions for both influx and egress. The All-turbinate influx showed a significant Aβ subgroup main effect  and an Aβ subgroup by time interaction (Fig. 5D). The All-turbinate egress also showed an Aβ main effect and Aβ subgroup by time interaction (Fig. 5E). Out of concern that the thresholded time segments have a potential risk of under- or over-estimation of tracer accumulation in the influx and egress periods, we compared a 2 min threshold against the 5 min threshold in the separation of the Aβ subgroups. The results thresholded at 2 min were essentially unchanged (Supplementary Table 4).

To further characterize the effects of Aβ subgroup on tracer influx and egress we normalized tracer egress by influx. The ratio results for the All-turbinates was elevated reflecting a disproportionate reduction in influx relative to tracer egress (Fig. 5F). This effect was not found for the LOF, which demonstrated a matched influx and egress (Fig. 5C). A similar result was found for the All-turbinates 60 min t75% clearance time which was greater in Aβ+ subjects (Supplementary Table 2). Overall, Aβ+ subjects show reduced tracer input to brain and nose and for both regions longer times to clear.

The association between tracer egress from LOF and from the All-turbinates further supported the hypothesized brain and nose relationship. Over the entire sample, the association between tracer egress from LOF and the All-turbinates was significant (Spearman correlation *r* = 0.61, *p* = 0.002). Moreover, this relationship appears to be driven by the Aβ negative individuals (*r* = 0.68, *p* = 0.005), it was not observed in the Aβ + individuals (*r* = 0.28, *p* = 0.50).

A more granular evaluation of the influx and egress TACs across the cribriform plate, superior, middle, and inferior turbinates) additionally supports the observed impairment of tracer kinetics within Aβ + subjects (Supplementary Fig. 1, Supplementary Table 2). Together, these data demonstrate in individuals with brain Aβ depositions, impairments in tracer egress from the brain and impairments in influx to and egress from the nasal turbinates. Overall, the results suggested a primary fluid kinetic impairment in the amyloid positive brain.

### Tracer egress from the carotid artery

We evaluated the potential for tracer delivered to the brain via the carotid artery to contribute to the observed brain and nose kinetic impairments. In the first few minutes following PET tracer administration the arterial blood concentration is directly related to blood flow and volume [41]. In our sample, all subjects exhibited carotid artery tracer peaks in under 30 s (see Fig. 3B). When assessing the effects of brain amyloid on the 0-60 min carotid artery TAC, we observed a significant main effect of Aβ subgroup (F(1,22) = 12.08; *p* = 0.0021), such that Aβ + individuals had a lower tracer concentration. There was no Aβ subgroup by time interaction.

Interestingly, for the tracer influx (0-30 s) to the carotid, there was neither an Aβ subgroup main effect nor an interaction effect observed (Supplementary Fig. 2A-B). This result suggests that the heart to carotid tracer delivery was not different by Aβ subgroup but the tracer going from carotid to brain was reduced in concentration in the Aβ + subgroup ***(***Supplementary Fig. 2C-D). The carotid egress period reflects mixed and recirculated blood from body and brain. As such, these data suggest reduced recirculated tracer available to brain in the Aβ + subgroup. We speculate that this effect contributes to the observed brain influx reduction (trend). In contrast, neither the jugular vein nor the temporalis muscle show significant Aβ subgroup related influx or egress effects (*p* > 0.05).

### Nasal and brain butanol clearance time in ApoE subgroups

Given a disproportionately high prevalence of ApoE Ɛ4 carriers in the Aβ + subgroup, and the potential for ApoE-driven neuroinflammation [42] as well as other contributions to CSF clearance [43], we assessed the TAC of [1-^11^C]-Butanol stratified by ApoE Ɛ4 status. No significant brain or nasal pathway clearance differences were observed between Ɛ4 carriers and non-carriers by Repeated Measures Two-Way ANOVA or the t75% (Supplementary Table 3).

## Discussion

### The relationship between brain and nasal turbinate kinetics

This paper introduces [1-^11^C]-Butanol as a novel PET tracer for *in-vivo* assessment of fluid clearance dynamics through the brain, nasal turbinates, carotid artery, and jugular vein. [1-^11^C]-Butanol is a freely diffusible tracer with a molecular weight of 74 Da that does not bind to brain and demonstrates a tissue and blood-brain-barrier permeability greater than water [44, 45]. Prior work has revealed the metabolic fate and modeling of [1-^11^C]-Butanol in clinical applications using Butanol as a blood flow agent [41, 46]. Capitalizing on these properties, we initially assessed the relationship between the brain and nasal turbinates for tracer concentration and kinetics over 60 min. For the entire cohort, the results showed significant concentration and time-dependent correlations between brain and nose. This effect supported the brain to nose communication hypothesis.

### The effect of amyloid on brain and nasal turbinate tracer influx and egress

Subsequently, we evaluated the effects of brain Aβ positivity on tracer influx and egress. We found that Aβ + individuals, when compared with Aβ- subjects, have lower tracer concentrations over 0-60 min in both the brain and nasal turbinates. We reasoned this Aβ subgroup difference could be driven by reduced tracer influx to and/or egress from the Aβ + brain. To address this question, based on prior knowledge of the time course of blood borne PET tracer delivery, we created influx and egress periods.

The Repeated Measures ANOVAs show that egress from brain and nose were consistently reduced in the Aβ + subgroup. Tracer influx was significantly lower for the nose (*p* < 0.05), possibly reflecting a reduced brain output (*p* = 0.089). Further, across all samples, correlation analysis showed that the egress AUCs from brain and nose were significantly correlated (Spearman correlation *r* = 0.61, *p* = 0.002). Together, these preliminary findings suggest that reduced egress of tracer from the brain may contribute to the reduced influx to the turbinates.

Adding to this complex picture, it remains unknown to what extent a reduction of tracer influx to brain drives the observed reduced brain and turbinate egress effects in Aβ + participants. Out of concern that the thresholded time segments have a potential risk of under- or over-estimation of tracer accumulation in the influx and egress periods, we compared a 2 min threshold against the 5 min threshold in the separation of the Aβ subgroups. The results were essentially unchanged (see Supplement Table 4).

### Interpretation and concerns

Overall, our results support the hypothesis that a brain fluid egress pathway includes the nose, but many uncertainties remain. Importantly, Butanol has yet to be validated as a CSF or interstitial fluid biomarker. Further, direct validation that human nasal fluids carry Aβ and other brain proteins, is limited [47].

Our data shows that brain Aβ impacts the clearance of fluid (presumably CSF) from brain and that the effect is detected in the nasal turbinates. Recent evidence indicates the turbinates may function as a broad inflammatory surveillance mechanism [48, 49] suggesting that the nose and its lymphatic architecture have a unique capacity to regulate the clearance of brain derived proteins. However, one cannot rule out alterations in the nasal mucosa secondary to Aβ-related inflammatory effects having an impact on nasal turbinate tracer dynamics.

Among the control regions examined in this study, which included the carotid artery, jugular vein, and temporalis muscle, only the carotid demonstrated reduced egress in the Aβ + subgroup. This result for the carotid potentially reflects lower levels of recirculating tracer due to an impaired CSF egress from brain or increased recycling time due to vascular pathology [50]. As such, we propose that the brain and nasal effects in Aβ + subjects are anatomically selective, but more tissues need to be sampled. Overall, our data support the interpretation of a nasal CSF drainage pathway in humans and underscore an association between brain and nasal clearance adversely impacted in the presence of brain amyloidosis.

### The glymphatic perspective

Traditional understanding of CSF clearance to the periphery has emphasized the role of arachnoid granulations, responsible for absorbing subarachnoid CSF and transporting it into venous drainage [16]. However, contemporary studies have revised this perspective, indicating that this pathway is less significant than others [51]. Emerging evidence shows CSF drainage relies, in part, on the intracranial paravascular glymphatic system [6, 7]. This drainage is aquaporin 4 regulated along perivascular and lymphatic vessels of the dura mater [52, 53], pushing fluids and soluble waste products into the venous circulation. Specifically in murine models, increased Aβ plaque deposition is associated with reduced glymphatic clearance [54, 55]. Moreover, murine studies demonstrated that impairing the glymphatic system results in an increase in amyloid deposition throughout the brain [5, 6, 56–58]. It is possible, given these findings, that our observed changes in Butanol influx and egress reflect Aβ associated disruption in glymphatic function.

#### Histology and magnetic resonance imaging in brain clearance

Without exception, non-human mammals demonstrate a robust clearance pathway spanning from the subarachnoid space to cribriform plate and to nasal turbinates in sheep [59], rats [60], dogs [61], rabbits [62], and mice [63]. Johnston et al. [20]. first identified in human cadavers a potential cribriform plate CSF drainage pathway using cisterna magna injections. As we previously demonstrated *in-vivo* using a PET tau tracer, nasal turbinate clearance was reduced in AD subjects and associated with brain amyloid deposits [1]. There is also imaging [64] and neuropathologic evidence in AD that the nasal turbinates and olfactory bulb accumulate tau, while amyloid accumulations in the turbinates are limited [65]. Nevertheless, the in-vivo assessment and interpretation in humans remains preliminary.

In-vivo magnetic resonance (MR) contrast examinations of CSF drainage through the nasal turbinates and cribriform plate have produced somewhat conflicting reports. For instance, serial T1-weighted and T2W-fluid attenuated inversion recovery imaging over 39 h following intrathecal administration of gadodiamide, showed clearance in the meningeal lymphatics, peri-optic pathway, and olfactory pathway including the superior, middle, and inferior turbinates [66]. On the other hand, Melin et al. observed gadobutrol near the cribriform plate in sequential MR scans over 48 h, however only a portion of their participants showed contrast in the turbinates or the nasal septum [21]. It remains unclear whether these different outcomes are influenced by MR time sampling; the physiochemical properties of the specific gadolinium contrast agents [67], gadolinium-based tracers are an order of magnitude larger (MW: 604 Da) than the Butanol PET radiotracer (MW: 74 Da), and Butanol is a freely diffusible lipophilic tracer in contrast to the lipophobic profile of gadolinium-based tracers. As such, MRI and PET examinations are not sampling the same anatomical clearance pathways [68]. Overall, combined MRI and PET validations [69] are needed to identify optimal approaches to estimating CSF to blood and brain to nose clearance. Nevertheless, it remains undemonstrated whether impaired CSF clearance in humans leads to the aggregation of Aβ fibrils or vice versa.

### Study limitations

Our preliminary and cross-sectional results support the utilization of the lipophilic, highly permeable, and nonbinding [1-^11^C]-Butanol PET tracer in assessment of brain and nose fluid dynamics, however, additional validations are needed to establish its role as a CSF biomarker. Replication in larger sample sizes and longitudinal comparisons are crucial for clinical validation.

A limitation of any PET imaging study remains the half-life of the tracer utilized, the camera sensitivity, and its resolution. This is especially relevant in consideration of the sampling of smaller regions of interest, like the carotid artery, where 4-6 mm PET camera resolution, relatively small lumen size (4-7 mm), and low counts, add noise that can bias results. It is also a consideration in the sampling of nasal turbinates, which provide a large target but also can include relatively large volumes of air space in relation to total tissue volume, thus impacting PET tracer recovery.

Of further interest, the PET research subjects were scanned in the supine position, which has been associated with a higher venous pressure in jugular veins than sitting position [70]. As such, the optimal position for human scanning remains unknown. Recent insights into the biomechanics and modeling of glymphatic transport in rodent models suggest that lateral and supine positions are ideal for fluid transport [71]. It remains possible that alternative biomechanical parameters in humans may influence the dynamics and kinetics of CSF circulation. Further research is needed to clarify the relevance of these observations to human physiology.

Moreover, it is unknown whether nasal olfactory function, which is clinically observed to be affected in AD, is affected by amyloid or by reduced nasal clearance. While our study excluded significant olfactory disease, unfortunately we did not examine olfactory function. In murine models, ablation of the olfactory sensory nerves and turbinates has been shown to impede CSF drainage [72]. In humans, impaired olfaction has been linked to the progression from amnestic mild cognitive impairment to Alzheimer’s disease [73], but any association with nasal CSF dynamics remains unknown.

## Conclusion

Our human data are consistent with findings from the murine literature that interstitial fluids are cleared from the brain through the cribriform plate and nasal turbinates. Specifically, we observe temporally correlated tracer influx and drainage between brain, cribriform plate, and nose. Further, subjects with brain amyloid plaque depositions demonstrate reduced tracer influx to and delayed clearance from both brain and nasal turbinates. This effect was most sensitively observed in the nasal compartment. While our results support the hypothesis that CSF enters the nasal turbinates, the amount and precise fluid composition remains unclear. Therefore, it is uncertain whether the correlated clearance from brain and nasal turbinates are a direct result of brain Aβ deposits impairing CSF egress from the brain. This is an area that warrants further validation of fluid composition and the effects of Aβ on membrane permeability.

### Electronic supplementary material

Below is the link to the electronic supplementary material.

Supplementary Material 1

## Data Availability

The datasets used and/or analyzed during the current study are available from the corresponding author on reasonable request.

## Abbreviations

AD: Alzheimer’s disease
CDR: Clinical Dementia Rating Scale
CRAFT: Craft Story 21 Recall-Delay
CSF: Cerebrospinal fluid
FBB: (^18^F)-labeled Florbetaben
GDS: Global Deterioration Scale
IQR: interquartile range md:median
MRI: Magnetic Resonance Imaging
MW: Molecular Weight
PET: Positron emission tomography
PiB: (^11^C)-labeled Pittsburgh Compound-B
RAVLT: Rey Auditory Verbal Learning Total Recall-Delayed
SUV: Standardized uptake value
