# Supplementary material for: [1-11C]-Butanol Positron Emission Tomography reveals an impaired brain to nasal turbinates pathway in aging amyloid positive subjects
Source: Fluids Barriers CNS. 2024 Apr 2;21:30. doi: 10.1186/s12987-024-00530-y (PMC10985958; doi:10.1186/s12987-024-00530-y)
Supplement: Supplementary file 1 — Supplementary Material 1 [file 12987_2024_530_MOESM1_ESM.docx]

| **Demographic Measures** | | | | | |
| --- | --- | --- | --- | --- | --- |
|  | **ApoE e4- (n=15)** | **ApoE e4+ (n=9)** | **Statistical Test** | **p** |  |
| Age - Mean (SD) [Range] | 77. 5 (6.4) [66-86] | 71.2 (4.6) [65-78] | Mann-Whitney | **0.02** |  |
| Gender – Female/Male (%) | 8 (53.3)/7 (46.7) | 6 (66.7)/3 (33.3) | χ2 | 0.52 |  |
| Weight (kg)–Mean (SD) [Range] | 74.9 (16.1) [54-121] | 64.6 (13.9) [48-94] | Mann-Whitney | 0.095 |  |
| Beta-Amyloid Positive (%) | 1 (6.7) | 7 (77.8) | χ 2 | **0.001** |  |
| **Cognitive Measures** | | | | | |
| CDR md (IQR) | 0 (0) | 0 (0) | Mann-Whitney | 0.99 |  |
| GDS md (IQR) | 2 (1.0) | 2 (0.5) | Mann-Whitney | 0.84 |  |
| CRAFT md (IQR) | 14 (6.0) | 16 (6.0) | Mann-Whitney | 0.32 |  |
| RAVLT md (IQR) | 8 (7.0) | 11 (7.0) | Mann-Whitney | 0.43 |  |

**Supplementary Table 1.** **Demographic and cognitive performance across APOE allele subgroups.**

Abbreviations: Standard Deviation (SD); kilograms (kg); Interquartile range (IQR), median (md), Clinical Dementia Rating (CDR), Global Deterioration Scale (GDS), Rey Auditory Verbal Learning Total Recall-Delayed (RAVLT), Craft Story Recall-Delayed (CRAFT). Significant differences (p ≤0.05) denoted by bolded values.

**Supplementary Figure 1.** **PET Butanol Input and Egress in the nasal space.** The impact of brain amyloid positivity on PET Butanol within the influx and egress for cribriform plate (A-D), superior turbinate (E-H), middle turbinate (I-L) and inferior turbinate (M-P). Aβ- individuals are displayed in black, and Aβ+ in red throughout. Error is represented as standard error of the mean. The analyses were performed on interpolated values across time to avoid biasing results from oversampling of more frequent early intervals.

Across all regions, the SUV time activity curve is shown across influx (0-5min) and egress (5-60min). The Aβ subgroup AUC differences for the egress and influx and their ratio was evaluated with Mann-Whitney tests.

**Cribriform plate:** The Aβ subgroup AUC differences were not significantly different for influx (B), for egress (D), nor for the egress/influx ratio.

**Superior Turbinate:** The Aβ subgroup AUC differences were significant for influx (F) (p <0.05) and for egress (H) (p <0.05) where significantly lower AUCs are found in Aβ+ individuals. No significant difference was observed in egree/influx ratio.

**Middle Turbinate:** The Aβ subgroup AUC differences were significant for influx (J) (p <0.05) and for egress (L) (p <0.05) where significantly lower AUCs are found in Aβ+ individuals. A trend in egress/influx ratio was observed (p=0.0926), with an elevated egress/influx ratio in Aβ+ individuals.

**Inferior Turbinate:** The Aβ subgroup AUC differences were significant for influx (N) (p <0.05) and for egress (P) (p <0.05) where significantly lower AUC are found in Aβ+ individuals. A significantly elevated egress/influx ratio was found in Aβ+ individuals (p=0.0057).

**Supplementary Figure 2.** **PET Butanol Input and Egress in control regions.** The effects of brain amyloid positivity on PET Butanol influx and egressfor carotid artery (A-D), jugular vein (E and H), and temporalis muscle (I-L). Aβ- individuals are displayed in black, and Aβ+ in red. Error is represented as standard error of the mean.

In consideration that all subjects demonstrated arterial blood peaks in less that 30 sec, the carotid influx and egress threshold was adjusted to 30 seconds. Similarly, as with the LOF and All turbinates, a main effect of Aβ group, and Aβ group by time interaction was evaluated by two-way ANOVA for both influx and egress in the carotid. The jugular vein and temporalis muscle were evaluated using the 5-minute threshold used in the other regions. The AUC for both influx and egress and their ratio were evaluated with Mann-Whitney tests.

The carotid demonstrated a main effect of Aβ group on carotid egress (D)(F(1, 22) = 12.57 p<0.0018), such that there was significantly lower tracer concentration in Aβ+ individuals. This was not observed for tracer influx (B). No significant difference was observed in AUC for influx or egress/influx ratio.

Neither the jugular nor temporalis muscle demonstrated influx or egress or ratio effects (p>.05).

| **Region of Interest** | **t75% (seconds) by Aβ subgroup**  Median (SD) | | **p-value** | **FDR Adjusted p-value** |
| --- | --- | --- | --- | --- |
|  | Aβ- (n=16) | Aβ+ (n=8) |  |  |
| Cribriform Plate | 2540 (85.1) | 2630 (39.6) | 0.023* | 0.046* |
| All-turbinates | 2510 (59.6) | 2595 (64.1) | 0.011* | 0.046* |
| Superior Turbinate | 2535 (58.4) | 2570 (56.3) | 0.243 | 0.278 |
| Middle Turbinate | 2470 (67.9) | 2605 (80.2) | 0.013* | 0.046* |
| Inferior Turbinate | 2515 (84.2) | 2590 (97.5) | 0.020* | 0.046* |

**Supplementary Table 2**. **Median Butanol t75% nasal clearance times (seconds) by Aβ subgroup.** Regional tracer times (seconds) to clear 75% of the tracer by Aβ subgroup. The regional nasal pathway clearance time differences between Aβ subgroups are unchanged after adjustment for false discovery rate (FDR). Shown are the unadjusted and FDR adjusted p-values. * p-values ≤ .05.

| **Region of Interest** | **t75% (seconds) by ApoE subgroup** | | | **p-value** | **Adjusted p-value** |
| --- | --- | --- | --- | --- | --- |
|  | e4+  (n=9) | e4-  (n=15) | (e4+) - (e4-) |  |  |
| LOF | 2663 | 2669 | -6 | 0.903 | 0.9030 |
| All-Turbinates | 2580 | 2510 | 70 | 0.100 | 0.3803 |
| Superior Turbinate | 2550 | 2540 | 10 | 0.720 | 0.8100 |
| Middle Turbinate | 2580 | 2480 | 100 | 0.339 | 0.5837 |
| Inferior Turbinate | 2570 | 2530 | 40 | 0.169 | 0.3803 |
| Cribriform Plate | 2620 | 2550 | 70 | 0.083 | 0.3803 |
| Temporalis Muscle | 2700 | 2680 | 20 | 0.403 | 0.5837 |
| Carotid Artery | 2549 | 2526 | 23 | 0.454 | 0.5837 |
| Jugular Vein | 2370 | 2470 | -100 | 0.151 | 0.3803 |

**Supplementary Table 3. Median regional Butanol t75% clearance time (seconds) by ApoE carrier status.** None of the p-values achieved significance. Adjusted p-values reflect corrections for false discovery rate.

|  | **Comparing two Tracer Thresholds on the discrimination of Aβ Subgroup** | | | |
| --- | --- | --- | --- | --- |
| **Region of Interest** | **Influx 0-2min** | **Egress 0-2min** | **Influx 0-5min** | **Egress 0-5min** |
| Lateral Orbitofrontal | F(1,22)=1.33, p=0.2606 | F(1,22)=4.982, **p=0.0361** | F(1,22)=3.16, p=0.0892 | F(1,22)=4.641, **p=0.024** |
| All-Turbinates | F(1,22)=7.499, **p=0.0120** | F(1,22)=11.70, **p=0.0024** | F(1,22)=10.24, **p=0.0041** | F(1,22)=11.36, **p=0.0028** |

**Supplementary Table 4. Comparison of 2min vs. 5min thresholds on influx and egress predictions of Aβ subgroup.** To address concerns about the effect of an arbitrary 5min threshold on charaterization of influx and egress predictors of Aβ subgroup we examined and compared a 2min threshold. Our findings demonstrate comparable results for LOF and All-turbinate for influx and egress. Significant main effects (p<0.05) are bolded.
